# Lipopolysaccharide Impedes Bone Repair in *FcγRIIB*-Deficient Mice

**DOI:** 10.3390/ijms242316944

**Published:** 2023-11-29

**Authors:** Sirikanda Jantaboon, Nithidol Sakunrangsit, Parichart Toejing, Asada Leelahavanichkul, Prapaporn Pisitkun, Matthew B. Greenblatt, Sutada Lotinun

**Affiliations:** 1 Interdisciplinary Program of Physiology, Graduate School, Chulalongkorn University, Bangkok 10330, Thailand; sirimdmd@gmail.com; 2 Center of Excellence in Skeletal Disorders and Enzyme Reaction Mechanism, Department of Physiology, Faculty of Dentistry, Chulalongkorn University, Bangkok 10330, Thailandlookplanoi_@hotmail.com (P.T.); 3 Division of Immunology, Department of Microbiology, Faculty of Medicine, Chulalongkorn University, Bangkok 10330, Thailand; aleelahavanit@gmail.com; 4 Division of Allergy, Immunology, and Rheumatology, Department of Medicine, Faculty of Medicine, Ramathibodi Hospital, Mahidol University, Bangkok 10400, Thailand; beepisitkun@gmail.com; 5 Department of Pathology and Laboratory Medicine, Weill Cornell Medicine and Research Division, Hospital for Special Surgery, New York, NY 10065, USA; mag3003@med.cornell.edu

**Keywords:** lipopolysaccharides, inflammation, osteoimmunology, bone regeneration, systemic lupus erythematosus

## Abstract

Chronic inflammation contributes to the development of skeletal disorders in patients with systemic lupus erythematosus (SLE). Activation of the host immune response stimulates osteoclast activity, which in turn leads to bone loss. Regenerating bone in the inflammatory microenvironments of SLE patients with critical bone defects remains a great challenge. In this study, we utilized lipopolysaccharide (LPS) to imitate locally and systemically pathogenic bacterial infection and examined the bone regeneration performance of LPS-associated mandibular and tibial bone regeneration impairment in *FcγRIIB^−/−^* mice. Our results indicated that a loss of *FcγRIIB* alleviates bone regeneration in both mandibles and tibiae. After LPS induction, *FcγRIIB^−/−^* mice were susceptible to impaired fracture healing in tibial and mandibular bones. LPS decreased the mineralization to collagen ratio in *FcγRIIB^−/−^* mice, indicating a mineralization defect during bone repair. An osteoblast-associated gene (*Col1a1*) was attenuated in *FcγRIIB*-deficient mice, whereas *Bglap, Hhip,* and *Creb5* were further downregulated with LPS treatment in *FcγRIIB^−/−^* mice compared to *FcγRIIB^−/−^* mice. *Alpl and Bglap* expression was dcreased in osteoblasts derived from bone chips. An osteoclast-associated gene, *Tnfsf11/Tnfrsf11* ratio, ewas increased in LPS-induced *FcγRIIB^−/−^* mice and in vitro. Furthermore, systemic LPS was relatively potent in stimulating production of pro-inflammatory cytokines including TNF-α, IL-6, and MCP-1 in *FcγRIIB^−/−^* mice compared to *FcγRIIB^−/−^* mice. The levels of TNF-α, IFN-β, IL-1α, and IL-17A were increased, whereas IL-10 and IL-23 were decreased in *Fcγ*RIIB^−/−^ mice treated locally with LPS. These findings suggest that both local and systemic LPS burden can exacerbate bone regeneration impairment, delay mineralization and skeletal repair, and induce inflammation in SLE patients.

## 1. Introduction

Lupus, also called systemic lupus erythematosus (SLE), is a complex autoimmune disease characterized by immune complex formation and autoantibody production that causes inflammation in multiple tissues such as cartilage and bone [1]. Anti-double-stranded deoxyribonucleic acid (dsDNA) antibodies are a pivotal diagnostic marker and can be used as a criterion to classify SLE. Approximately 60–90% of all SLE patients exhibit positive anti-dsDNA antibodies, and the presence of anti-dsDNA antibodies in serum is associated with SLE manifestations and the progression of lupus nephritis [2]. IgG is the most abundant isotype of autoantibodies that have the capacity to activate Fc gamma receptors (FcγRs) on B cells, neutrophils, dendritic cells, and macrophages. Numerous cellular effector functions are engaged via the cross-linking of FcγRs by IgG-immune complexes, including phagocytosis, degranulation, antibody-dependent cellular toxicity, and massive cytokine production.

FcγRs have been classified into three different groups in humans; FcγRI, FcγRII, and FcγRIII. Additionally, FcγRII and FcγRIII have both isoform A and B forms that carry out disparate functions. FcγRII and FcγRIII are low affinity receptors that mostly bind IgG-immune complexes, while FcγRI is a high affinity receptor that binds monomeric IgG as well as IgG-immune complexes [3]. The activating receptors are FcγRI, FcγRIIA, FcγRIIIA, and FcγRIIIB. FcγRI and FcγRIIIA are made up of a γ chain with three and two Ig-like domains, respectively, that are coupled to a cytoplasmic signaling component. The immunoreceptor tyrosine-based activation motif (ITAM) of the γ chain is a key mediator of intracellular signaling. FcγRIIA is a single chain receptor with an ITAM motif in its cytoplasmic tail. FcγRIIB is a structurally similar inhibitory receptor to FcγRIIA, but it possesses an immunoreceptor tyrosine-based inhibitory motif (ITIM) in the cytoplasmic domain. FcγRIIB has been unveiled to play a significant negative regulatory role in Fc receptor activation.

The functions of FcγRs in experimental lupus have been a prior focus of study, and it is now evident that activating FcγRs is required for disease development and progression. *FcγRI/III* double-deficient mice are protected from nephrotoxic nephritis [4]. The absence of *FcγRIIB* in mice increases susceptibility to inflammatory autoimmune diseases including SLE. Recently, it was reported that *FcγRIIB*-deficient mice display increased severity of immunological disorders such as collagen-induced arthritis (CIA) [5] and inducible alveolitis [6]. Current studies have demonstrated that *FcγRIIB^−/−^* mice display anti-dsDNA autoantibodies and have increased B220^low^CD138^+^ cells, indicating an active SLE-like disease [7]. Six-month-old *FcγRIIB* knockout mice exhibited cancellous and cortical bone osteopenia, elevated osteoclastogenesis, and hemolytic anemia [7,8,9]. These findings highlight the role of FcγRs in the pathophysiology of experimental lupus, which may also apply to lupus in humans. 

Genetic polymorphisms in the FcγRIIB promoter reduce receptor expression in mice and are likely to contribute to spontaneous SLE in a variety of animal models. In addition to enhanced autoimmune susceptibility, FcγRIIB impacts survival after bacterial infection through inducing bacterial clearance and plays a significant role in periodontitis, leading to alveolar bone loss [9]. SLE patients are at increased risk of periodontitis possibly as a result of a pathogenic immune response to oral bacteria and inflammation. Interestingly, there is an association between the FcγRIIB-232 I/T allele and clinical characteristics in SLE patients [10], and a functional variant of the 232T is associated with aggressive periodontal disease in Japanese patients [11]. Moreover, a study by Clatworthy et al. has indicated that the monocyte-derived macrophages are increased in the peripheral blood of patients with the SLE-associated FcγRIIB-232T genotype [12], which could lead to the identification of opportunities for using this receptor as a therapeutic target.

Bone loss has been reported to be associated with inflammatory diseases including rheumatoid arthritis, periodontitis, and SLE [1]. *Porphyromonas gingivalis* and *Escherichia coli* (*E. coli*) are identified as major periodontal pathogens. The pathogenesis of inflammatory periodontal diseases can be induced from lipopolysaccharide (LPS) derived from periodontal bacteria. *E. coli*-LPS activates toll-like receptor-4 (TLR-4) to induce inflammatory responses, including increased M1 polarization and pro-inflammatory cytokine production, unlike LPS from other bacteria (such as *P. gingivalis*), which binds to TLR-2 and is able to evade TLR-4 stimulation in macrophages [13]. LPS-induced bone loss models are frequently used to evaluate the interface between inflammation and osteopenia. To imitate local and systemic bacteria-induced defect site infections, we used *E. coli*-LPS in this study to verify the hypothesis that mice lacking *FcγRIIB* displayed aggravated inflammatory responses to infectious microenvironments which contribute to impaired bone regeneration. We found that *FcγRIIB* had an important role in LPS-induced inhibition of osteogenesis under inflammatory conditions in vivo and in vitro. The results showed that circulating inflammatory cytokines from mice with LPS could impair the skeletal repair process.

## 2. Results

### 2.1. LPS Increases Serum Calcium and Decreases Renal Function

**Figure 1:**
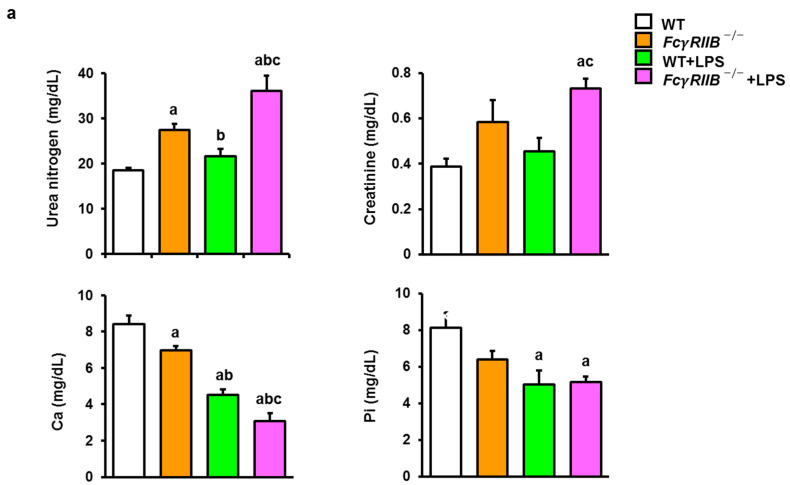

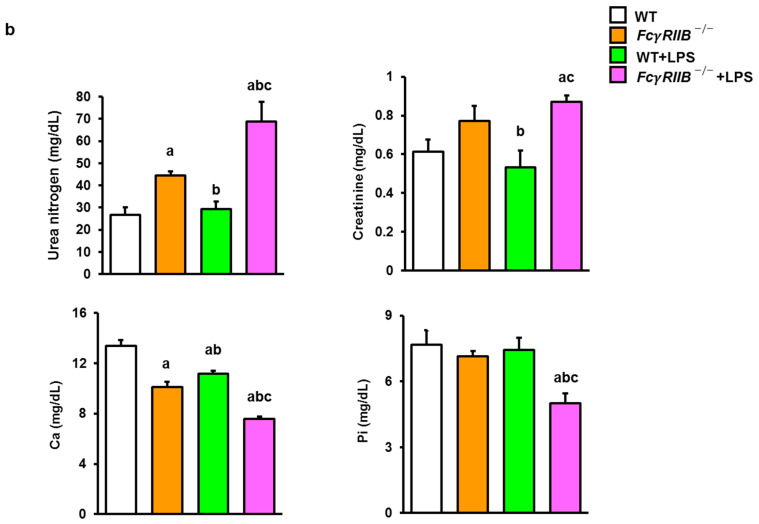
Local and systemic LPS increases serum calcium levels and reduces renal function. (**a**) Serum biochemistries of WT and *FcγRIIB^−/−^* mice treated with local LPS. (**b**) Serum biochemistries of WT and *FcγRIIB^−/−^* mice treated with systemic LPS. Data are mean ± SEM (*n* = 5–6). ^a^ *p* < 0.05 compared to WT; ^b^ *p* < 0.05 compared to *FcγRIIB^−/−^* mice; ^c^ *p* < 0.05 compared to WT+LPS.

It has been reported that a high phosphate and low calcium diet induced bone loss due to hyperparathyroidism [14]. High PTH levels are a potential cause of osteopenia and were observed in patients with SLE [15]. *FcγRIIB*^−/−^ mice had increased serum urea nitrogen levels, and local administration of LPS further enhanced serum urea nitrogen levels in *FcγRIIB*^−/−^ mice (Figure 1a). Serum creatinine was increased in *FcγRIIB*^−/−^+LPS mice compared to WT+LPS mice. Serum calcium concentration was decreased in all groups. Serum phosphorus was decreased in WT+LPS compared to WT controls. 

Ablation of *FcγRIIB* also increased serum urea nitrogen and decreased serum calcium levels (Figure 1b). Systemic LPS decreased serum calcium levels in WT mice when compared to WT controls. Meanwhile, statistical analysis showed that serum urea nitrogen and creatinine levels were significantly enhanced in *FcγRIIB^−/−^* mice with systemic LPS. Serum calcium and phosphorus levels were decreased in *FcγRIIB^−/−^*+LPS mice.

### 2.2. FcγRIIB^−/−^ Mice Have Delayed Mandibular Bone Regeneration That Is Further Exacerbated by Local LPS

**Figure 2:**
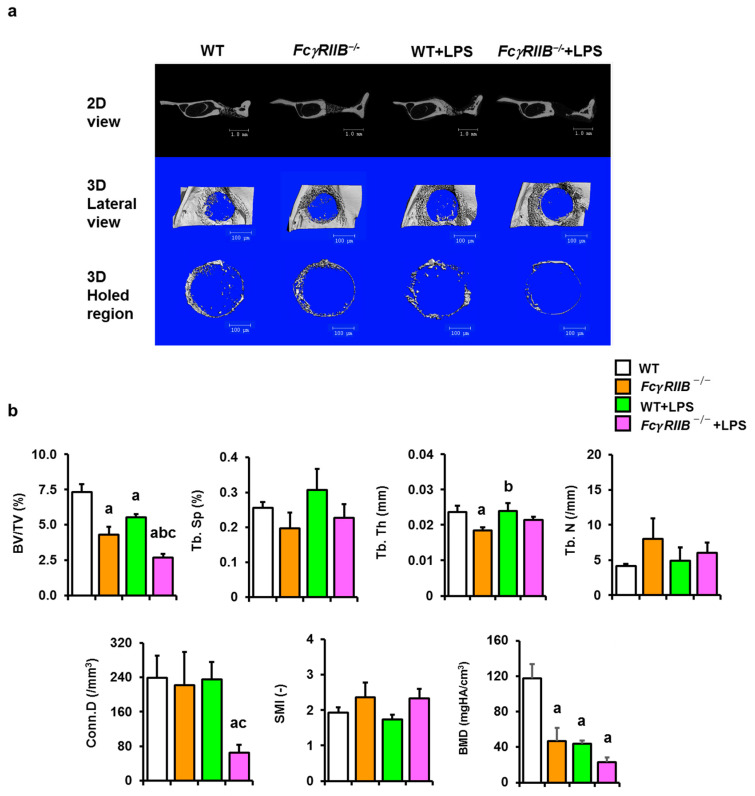
Local LPS worsens mandibular bone regeneration in *FcγRIIB^−/−^* mice. (**a**) 2D and 3D µCT reconstruction of drill-hole bone defects in the mandible of all groups. Scale bar = 100 µm. (**b**) Mandibular bone microarchitectural parameters. Cancellous bone volume/tissue volume (BV/TV), trabecular separation (Tb.Sp), trabecular thickness (Tb.Th), trabecular number (Tb.N), connectivity density (Conn.D), structure model index (SMI), and bone mineral density (BMD). Data are mean ± SEM (*n* = 5–6). ^a^ *p* < 0.05 compared to WT; ^b^ *p* < 0.05 compared to *FcγRIIB^−/−^* mice; ^c^ *p* < 0.05 compared to WT+LPS.

To investigate whether the deletion of *FcγRIIB* affected monocortical bone defect regeneration in mandibles and to evaluate the impact of *E. coli*-LPS administration-induced bone regeneration impairment in six-month-old male *FcγRIIB^−/−^* mice, µCT analysis was performed after inducing cortical drill-hole bone defects (Figure 2a). µCT analysis indicated that cancellous bone volume, trabecular thickness, and bone mineral density (BMD) were significantly decreased in *FcγRIIB^−/−^* mice (Figure 2b). Cancellous bone volume and connectivity density were decreased in the *FcγRIIB^−/−^*+LPS mice compared to WT+LPS controls. In WT mice with local LPS treatment, cancellous bone volume and BMD were significantly reduced. Attenuation of cancellous bone volume was observed in local LPS-induced *FcγRIIB^−/−^* mice compared to vehicle-treated *FcγRIIB^−/−^* comparators. Two-way ANOVA indicated no interaction between *FcγRIIB* and LPS.

### 2.3. FcγRIIB Deficiency Causes a Decrease in Tibial Bone Regeneration and Local LPS Impaired Bone Repair

**Figure 3:**
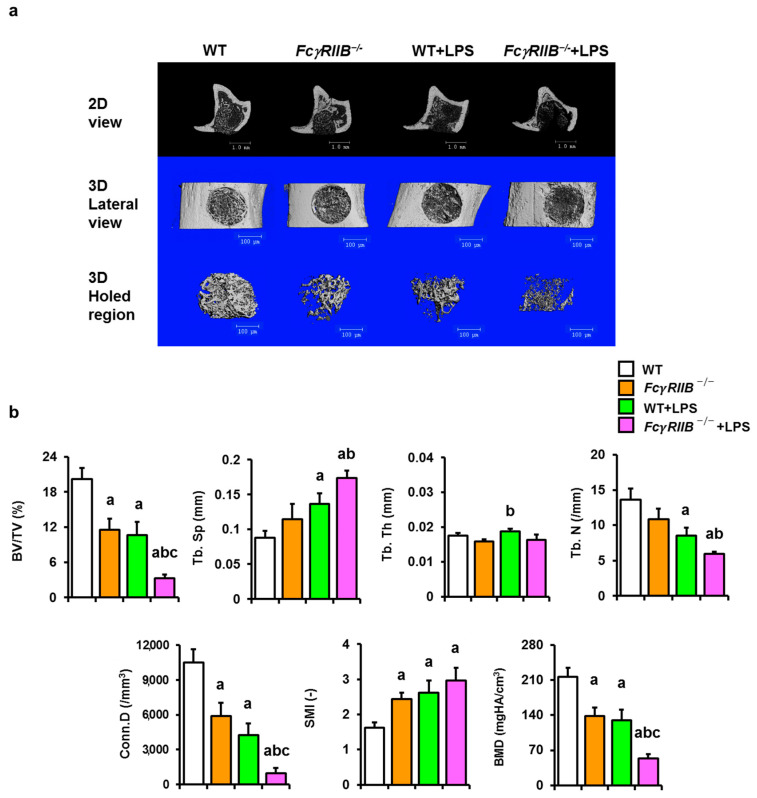
Local LPS impairs bone healing in the tibiae of *FcγRIIB^−/−^* mice. (**a**) 2D and 3D µCT reconstruction of drill-hole bone defects in the tibiae of all groups. Scale bar = 100 µm. (**b**) Tibial bone microarchitectural parameters. Cancellous bone volume/tissue volume (BV/TV), trabecular separation (Tb.Sp), trabecular thickness (Tb.Th), trabecular number (Tb.N), connectivity density (Conn.D), structure model index (SMI), and bone mineral density (BMD). Data are mean ± SEM (*n* = 6–8). ^a^ *p* < 0.05 compared to sham WT; ^b^ *p* < 0.05 compared to *FcγRIIB^−/−^* mice; ^c^ *p* < 0.05 compared to WT+LPS.

We also determined the bone microarchitecture in tibiae after local administration of LPS via the application of LPS on a collagen hemostat on a drill hole in *FcγRIIB^−/−^* mice (Figure 3a). Similar to the effects observed in mandibles, cancellous bone volume in *FcγRIIB^−/−^* mice was 43% less than that of WT controls. The connectivity density and BMD of the *FcγRIIB^−/−^* mice were 44 and 36% lower than those of the WT group, respectively, whereas the structural model index was increased (Figure 3b). The cancellous bone volume, trabecular number, connectivity density, and BMD of WT+LPS mice were less than those of the WT group. Trabecular separation and structural model index were increased in WT+LPS compared to WT controls. In *FcγRIIB^−/−^*+LPS mice, cancellous bone volume, connectivity density, and BMD were decreased compared to WT+LPS and *FcγRIIB^−/−^* mice. Two-way ANOVA indicated no interaction between *FcγRIIB* and LPS.

### 2.4. Systemic LPS Alleviates Skeletal Repair in Mice with FcγRIIB Ablation

**Figure 4:**
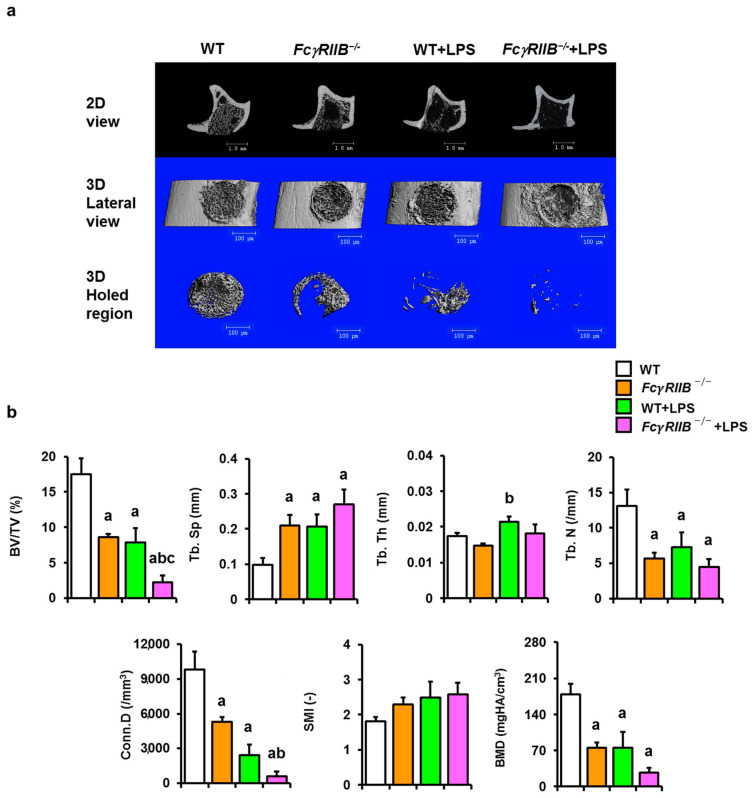
Systemic LPS increases susceptibility to impaired bone regeneration in FcγRIIB-deficient mice. (**a**) 2D and 3D µCT reconstruction of drill-hole bone defect in the tibiae of all groups. Scale bar = 100 µm. (**b**) Tibial bone microarchitectural parameters. Cancellous bone volume/tissue volume (BV/TV), trabecular separation (Tb.Sp), trabecular thickness (Tb.Th), trabecular number (Tb.N), connectivity density (Conn.D), structure model index (SMI), and bone mineral density (BMD). Data are mean ± SEM (*n* = 5–7). ^a^ *p* < 0.05 compared to WT; ^b^ *p* < 0.05 compared to *FcγRIIB^−/−^* mice; ^c^ *p* < 0.05 compared to WT+LPS.

To further characterize the cancellous bone microarchitecture in *FcγRIIB*^−/−^ mice post exacerbation of inflammation by systemic LPS treatment, µCT analysis was performed, which indicated that impaired bone regeneration was found in *FcγRIIB^−/−^* mice and that this was further impaired when these mice were administrated systemic LPS (Figure 4a). The microarchitecture of proximal metaphysis of tibiae showed that cancellous bone volume, trabecular number, connectivity density, and BMD were decreased and trabecular separation was increased in *FcγRIIB^−/−^* mice compared with WT littermates (Figure 4b). Systemic LPS treatment, a subcutaneous injection of LPS, worsened bone microarchitecture including cancellous bone volume, trabecular number, connectivity density, and BMD. The structural model index was not altered. These findings demonstrate that LPS administration systemically reduces the microarchitecture parameters of cancellous bones in *FcγRIIB^−/−^* mice. Two-way ANOVA indicated no interaction between *FcγRIIB* and LPS.

### 2.5. Local and Systemic LPS Reduces Skeletal Mineralization to Collagen Ratio

**Figure 5:**
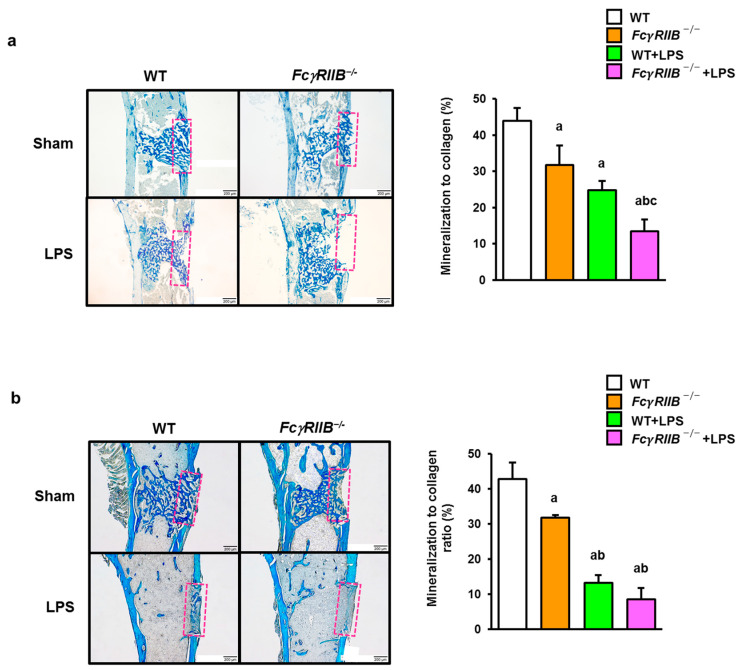
Local and systemic LPS decreases the mineralization to collagen ratio in tibial bone defects in *FcγRIIB^−/−^* mice. (**a**) Aniline blue staining showing the mineralization to collagen ratio in WT and *FcγRIIB^−/−^* mice treated with local LPS. (**b**) Aniline blue staining showing the mineralization to collagen ratio in WT and *FcγRIIB^−/−^* mice treated with systemic LPS. Dashed pink rectangles show the area of the mineralization to collagen ratio. Data are mean ± SEM (*n* = 5–6). ^a^ *p* < 0.05 compared to WT; ^b^ *p* < 0.05 compared to *FcγRIIB^−/−^* mice; ^c^ *p* < 0.05 compared to WT+LPS.

After bone microarchitectural analyses were performed, tibias were stained with aniline blue to demonstrate the collagen accumulation in the holed region. *FcγRIIB^−/−^* mice had a significant decrease in the mineralization to collagen ratio when compared to WT controls, indicating a delay in osteoid mineralization (Figure 5a). Local administration of LPS also repressed the mineralization to collagen ratio in WT groups. *FcγRIIB^−/−^* mice locally treated with LPS showed a significant decrease in the mineralization to collagen ratio compared to WT controls, *FcγRIIB*^−/−^, and WT+LPS mice. 

To further explore the mechanism of delayed bone regeneration in mice with systemic LPS, aniline blue staining was performed. We found that the mineralization to collagen ratio was reduced in *FcγRIIB^−/−^* mice (Figure 5b). Similarly, the aniline blue-positive tibial sections were decreased in WT+LPS mice. In *FcγRIIB*-deficient mice, the mineralization to collagen ratio was reduced compared to *FcγRIIB*^−/−^ mice after systemic LPS administration. Two-way ANOVA indicated no interaction between *FcγRIIB* and local and systemic LPS.

### 2.6. Decreased Osteoblast-Associated Genes and Increased Tnfsf11/Tnfrsf11b Ratio in FcγRIIB-Deficient Mice Treated with Systemic LPS

**Figure 6:**
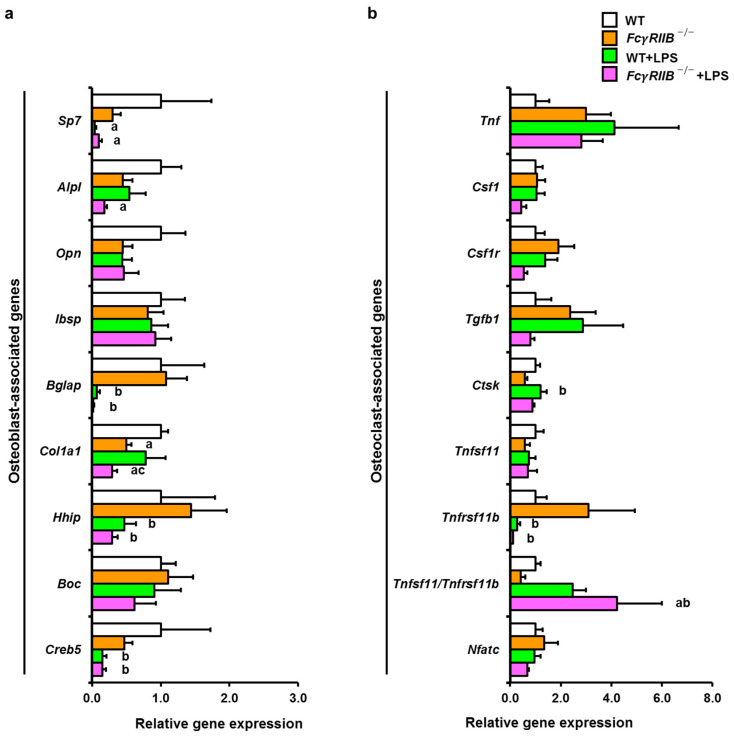
Systemic LPS downregulates osteoblast-associated genes and upregulates *Tnfsf11/Tnfrsf11b* expression. (**a**) qPCR analysis of osteoblast-associated genes in *FcγRIIB^−/−^* mice in the presence or absence of systemic LPS administration. (**b**) qPCR analysis of osteoclast-associated genes in *FcγRIIB^−/−^* mice in the presence or absence of systemic LPS administration. Data are mean ± SEM from three independent experiments (*n* = 6). ^a^ *p* < 0.05 compared to WT; ^b^ *p* < 0.05 compared to *FcγRIIB^−/−^*; ^c^ *p* < 0.05 compared to WT+LPS.

*FcγRIIB* deficiency caused a decrease in *Col1a1* expression compared to WT controls (Figure 6a). *Col1a1* was reduced in *FcγRIIB^−/−^*+LPS mice compared to WT+LPS mice. Osteoblast-associated genes such as *Sp7* were reduced in WT bones systemically treated with LPS. Systemic LPS decreased *Bglap*, *Hhip* (Hedgehog interacting protein), and *Creb5* (cAMP-response element binding protein 5) expression levels in *FcγRIIB^−/−^*+LPS mice compared to *FcγRIIB^−/−^* mice. The ratio of *Tnfsf11/Tnfrsf11b* mRNA expression was significantly increased in *FcγRIIB^−/−^*+LPS mice compared to *FcγRIIB^−/−^* mice (Figure 6b).

**Figure 7:**
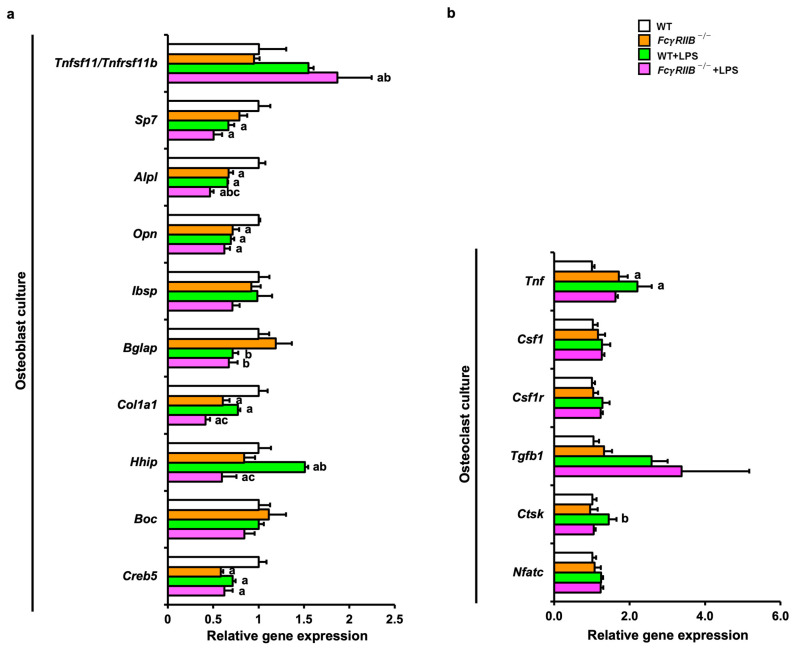
Systemic LPS treatment downregulates osteoblast-associated genes and upregulates *Tnfsf11/Tnfrsf11b* expression in vitro. (**a**) qPCR analysis of osteoblasts derived from bone chips in *FcγRIIB^−/−^* mice in the presence or absence of systemic LPS administration (*n* = 3–6). (**b**) qPCR analysis of osteoclasts derived from bone marrow macrophages in *FcγRIIB^−/−^* mice in the presence or absence of systemic LPS administration (*n* = 4–6). Data are mean ± SEM from three independent experiments. ^a^ *p* < 0.05 compared to WT; ^b^ *p* < 0.05 compared to *FcγRIIB^−/−^*; ^c^ *p* < 0.05 compared to WT+LPS.

We then determined gene expression profiles in osteoblasts derived from bone chips and osteoclasts from bone marrow macrophages. OB-related genes (*Alpl*, *Opn*, *Cola1*, *Creb5*) were decreased in *FcγRIIB^−/−^* mice (Figure 7a). After systemic LPS administration, expression levels of *Alpl* and *Bglap* were downregulated in *FcγRIIB*^−/−^ littermates compared to *Fcγ*RIIB^−/−^ mice. OC-related genes, *Tnfsf11/Tnfrsf11b*, were significantly increased in *Fcγ*RIIB^−/−^ mice treated with LPS compared to *FcγRIIB*^−/−^ mice (Figure 7b).

### 2.7. LPS-Induced Enhanced Inflammation in FcγRIIB^−/−^ Mice

**Figure 8:**
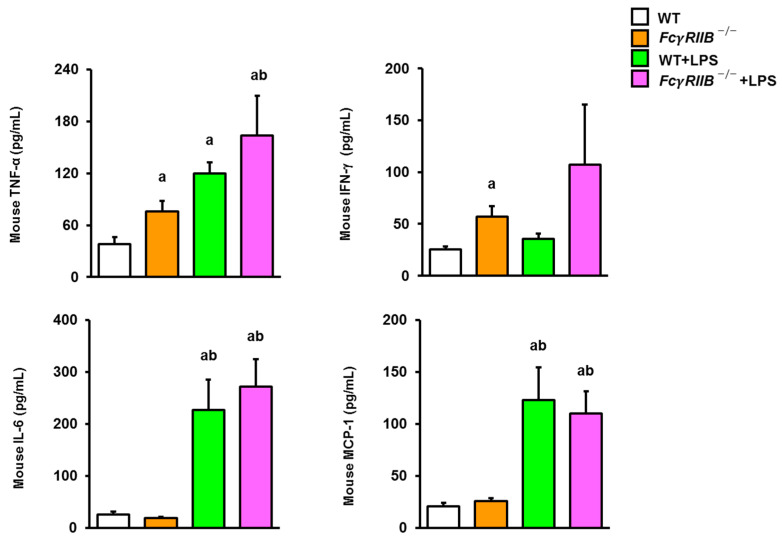
Systemic LPS treatment enhances serum levels of TNF-α, IL-6, and MCP-1 in *FcγRIIB^−/−^* mice. The concentration levels of pro-inflammatory cytokines including TNF-α, IFN-γ, IL-6, and MCP-1 are shown. Data are mean ± SEM (*n* = 5). ^a^ *p* < 0.05 compared to WT; ^b^ *p*< 0.05 compared to *FcγRIIB^−/−^*.

**Figure 9:**
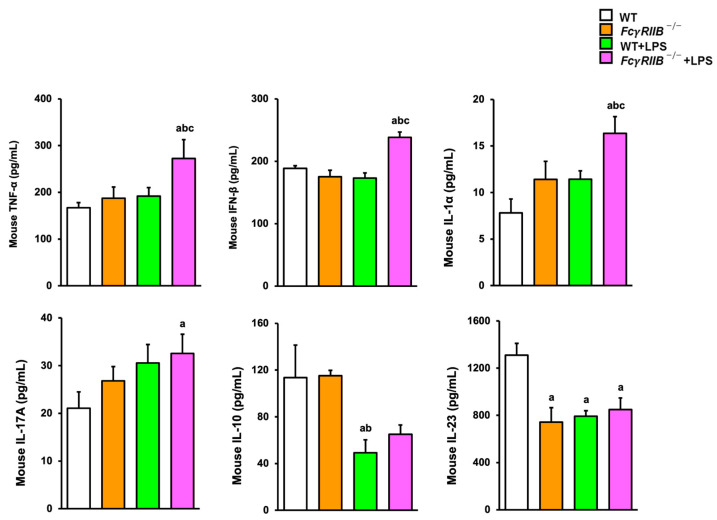
Local LPS treatment increases serum levels of TNF-α, IFN-β, IL-1α, and IL-7A and reduces serum levels of IL-10 and IL-23 in *FcγRIIB^−/−^* mice. Data are mean ± SEM (*n* = 5). ^a^ *p* < 0.05 compared to WT; ^b^ *p* < 0.05 compared to *FcγRIIB^−/−^*; ^c^ *p* < 0.05 compared to WT+LPS.

The pro-inflammatory cytokines TNF-α, IL-6, and MCP-1 play important roles in inflammation progression as a result of macrophage activation. To verify the effect of *FcγRIIB* deletion on the expression levels of several inflammatory mediators, we determined the levels of serum pro-inflammatory cytokines in *FcγRIIB^−/−^* mice treated with systemic LPS. A significant increase in serum concentrations of TNF-α and IFN-γ were detected in *FcγRIIB^−/−^* mice (Figure 8). The production levels of TNF-α, IL-6, and MCP-1 in WT mice systemically injected with LPS were higher than those in WT controls. Compared to WT and *FcγRIIB^−/−^* groups, enhanced serum TNF-α, IL-6, and MCP-1 levels were detected in *FcγRIIB^−/−^* mice after systemic LPS administration. Other cytokines, including IL-1α, IL-1β, IL-10, IL-12p70, IL17A, IL-23, IL-27, IFNβ, and GM-CSF, were not altered. For local LPS treatment, the levels of TNF-α, IFN-β, IL-1α, and IL-17A were higher in *FcγRIIB*^−/−^+LPS mice, whereas IL-10 and IL-23 were lower (Figure 9). These results suggest that LPS was strongly associated with inflammation, leading to impaired bone regeneration in SLE-like diseases.

## 3. Discussion

Chronic inflammation caused by SLE has a detrimental effect on bone microarchitecture, leading to low BMD and an increased risk of fragility fractures. The interaction of the immunoglobulin G Fc receptor and pro-inflammatory cytokines plays a significant role in the pathophysiology of SLE. In this study, we analyzed the impact of LPS as a model of local and systemic infection, such as periodontitis, on SLE-like disorders in the skeletons. *FcγRIIB^−/−^* mice exhibited a decrease in BMD and destruction of bone microstructures in both mandibles and tibiae. Serum levels of pro-inflammatory cytokines, including TNF-α and IFN-γ, were increased in these mice. Since LPS is one of the most critical pro-inflammatory stimuli mediating bacteria-induced bone loss, we evaluated the skeletal effects of LPS on the *FcγRIIB-*deficient mouse model of lupus. Our present study indicated that local and systemic administration of LPS ameliorates the cancellous bone microarchitecture with impaired cancellous bone volume, BMD, and mineralization to collagen ratio due to an increase in pro-inflammatory cytokines. To understand the molecular mechanism by which LPS affected inflammatory bone loss, the impact of *FcγRIIB* deficiency on LPS-induced signaling was investigated in systemic administration. Our findings indicated that ablation of *FcγRIIB* attenuated the expression of the osteoblast marker gene, *Col1a1*, and *Col1a1*, *Bglap*, *Hhip*, and *Creb5* were further downregulated by systemic LPS. The ratio of *Tnfsf11/Tnfrsf11b*, the osteoclast marker gene, was increased in *FcγRIIB^−/−^* mice with systemic LPS administration compared to *FcγRIIB^−/−^* mice. 

The expression of the B cell inhibitory receptor *FcγRIIB* is abnormally low in SLE, leading to inadequate suppression of autoantigen-mediated B cell receptor activation. Mice lacking *FcγRIIB* exhibit SLE on certain genetic backgrounds and its supplementation helps recovery from the disease [16,17]. *FcγRIIB* deficiency leads to decreased disposal of immune complexes, the breakdown of self-tolerance, and the inability to modulate inflammatory response. Japanese patients were susceptible to SLE and periodontitis as a result from not only the gain of function variants in *FcγRIIA* but also the loss of function variants in *FcγRIIB* [18]. Genetic variations in *FcγR*-induced IL-1β production are directly implicated in susceptibility to SLE [19]. Based on these findings and earlier studies, the imbalance of expression levels of inhibitory *FcγRIIB* is associated with spontaneous murine lupus [16]. Evidence from murine SLE, MRL/1 [20], and BXSB Yaa [21] models identified *FcγRIIB* as the key inhibitory receptor for several *FcγR*-mediated immune responses and *FcγRIIB*-mediated suppression of the aberrant cytokine milieu contributing to SLE phenotypes. 

Non-genetic variables that may be implicated in the development or progression of SLE include systemic exposure to bacterial components including LPS. LPS, a major component of the outer membrane of Gram-negative bacteria, is localized in the outer layer of the membrane and is in non-capsulated strains exposed on the cell surface. It can stimulate the release of inflammatory cytokines in various cell types, leading to an acute inflammatory response towards pathogens. LPS has been extensively used in inflammatory models as it mimics many inflammatory effects of cytokines. Consistent with other findings, systemic LPS stimulated the production of TNF-α, IL-6, and MCP-1 [22]. Local LPS decreased IL-10 and IL-23. IL-10 is a potent anti-inflammatory cytokine [23] and IL-23 inhibits osteoclastogenesis [24]. The release of these cytokines caused by LPS can aggravate altered bone turnover which favors bone resorption, leading to bone loss in both in vivo and in vitro studies. LPS also stimulates the granulocyte colony-stimulating factor, which suppresses bone formation while activating bone resorption [25].

LPS induces systemic inflammation via the infiltration of proteins and inflammatory cells into tissues. Flow cytometry indicates that CD11b-positive osteoclast precursors were increased after LPS injection. LPS stimulates TNF-α synthesis via NF-κB activation, leading to bone loss. It has been shown that early interventions of NF-κB stimulators, such as TNF-α, are able to induce osteogenesis in human adipose tissue-derived [26] and bone marrow-derived mesenchymal stem cells (MSCs) [27]. Neither TNF-α or LPS treatment have an effect on the expression of osteoblast-associated markers in bone marrow-derived MSCs without the presence of osteogenic supplements [27]. Furthermore, bacterial LPS can stimulate NF-κB, osteoclastogenesis, and osteolysis in the murine model [28]. Inactivating NF-κB abolishes LPS-induced excessive production of inflammatory mediators and ameliorates TNF-α-mediated osteogenic differentiation [26].

Periodontitis is the chronic inflammation and destruction of periodontal tissue which destroys the tooth-supporting apparatus. Gram-negative bacteria, including *Porphyromonas gingivalis* (*P. gingivalis*) and *Aggregatibacter actinomycetemcomitans,* were identified as major periodontal pathogens. They produce virulence factors that disturb host–microbe homeostasis. In addition to the microbial challenge, the progression of periodontitis is caused by local inflammation and overactivation of the host’s immune response which stimulates osteoclast activity, leading to mandibular bone loss. A potential correlation between periodontal and autoimmune diseases, including SLE, has been shown. The pathogenesis of inflammatory periodontal diseases can be induced from LPS derived from periodontal bacteria. *E. coli*-LPS can cause more systemic and local reactions than *P. gingivalis*-LPS in inducing inflammation [29]. LPS is a mediator of periodontal infection and macrophage activation. High serum LPS increases the ability of LDL to activate macrophages in patients with periodontitis [30]. Toll-like receptor 4 (TLR4), regulated by LPS, contributes to the loss of tolerances and patient disease activity in SLE [31].

Several cytokine network abnormalities have been discovered in SLE patients and murine lupus models. LPS administration into experimental animals causes massive secretion of TNF-α and other endogenous inflammatory mediators which are associated with clinical symptoms of SLE and periodontal diseases. The function of TNF-α in the pathogenesis of both diseases has been extensively investigated. NZB mice with *Tnf* deficiency had enhanced autoimmune responses and developed severe disease manifestations of SLE, such as glomerulonephritis [32]. In response to polyclonal and T helper stimuli, autoimmune reactions are accompanied with a regular spontaneous increase in serum concentration of anti-nuclear autoantibodies and hyperproliferating B cells that rapidly produce anti-dsDNA antibodies. Similarly, anti-dsDNA transgenic mice administered LPS displayed overproduction of anti-dsDNA autoantibodies and LPS-mediated TLR4 signaling, resulting in severe SLE-like syndromes through the hypersecretion of IL-10 and IFN-γ [33]. Given the observations of high serum TNF-α in active SLE and lupus nephritis, the TNF-α antagonist is still a promising therapy option for active SLE. 

LPS has been shown to be independent of *Tnfsf11/Tnfrsf11b* axis [34] but involves in TLR4/TNF-α signaling to activate osteoclastogenesis and bone resorption. Our study indicated that LPS induced bone regeneration impairment by increasing *Tnfsf11/Tnfrsf11b* and TNF-α in *FcγRIIB^−/−^*+LPS mice, compared to *FcγRIIB^−/−^* controls. The significance of *Tnfsf11/Tnfrsf11b* signaling has been determined by observing alveolar bone loss in *Tnfsf11* transgenic and *Tnfrsf11b* ^−/−^ mice. Severe bone resorption in the cortical areas of alveolar bone was observed in *Tnfrsf11b*^−/−^ mice but not in *Tnfsf11* transgenic mice [35]. An antimouse *Tnfsf11* antibody and risedronate inhibited alveolar bone resorption. A *Tnfsf11* binding peptide, WP9QY, suppressed alveolar bone loss by enhancing osteoblastogenesis and decreasing osteoclastogenesis in *Tnfrsf11b* ^−/−^ mice [35,36]. TLR4 activation by LPS exacerbated osteoclast function and activity by increasing *Tnfsf11* levels or directly promoting osteoclast progenitor cells [37], leading to impaired bone regeneration.

Systemic treatment with LPS can inhibit the bone repair process in vivo, while LPS at low concentrations stimulates osteoblast differentiation. Guo and colleagues [38] demonstrated that LPS significantly ameliorated osteoblast differentiation and induced apoptosis in MC3T3-E1 cells by activating the ERK1/2 and JNK pathway [38]. In contrast, Xu and colleagues reported that low concentrations of LPS induced osteoblast proliferation and accelerated fracture healing through the NF-κB signaling pathway in vitro [39]. The inhibitory effect of LPS on osteoblast differentiation by targeting the BMP2/Smad signaling pathway has been reported [40]. Osteoblast-associated genes including *Alpl*, *Ibsp*, *Col1a1*, and *Runx2* were downregulated by LPS, leading to a decrease in ALP activity and in vitro mineralization [40]. Creb5 stimulated osteoblast differentiation and the anabolic action of PTH was mediated by Creb transactivation of BMP 2 expression in bones [41]. It has been reported that the activation of Hh signaling involved in mineralization following osteoblastogenesis is achieved by modulating *COL1A1*, *SP7*, and *BGLAG* expression in human osteoblasts [42].

Hypocalcemia along with immunological amplification may be indicative of SLE activity. SLE patients with low serum calcium have increased peripheral cellular immunity [43]. Our results found sustained hypocalcemia in *FcγRIIB^−/−^* mice, and the concentration of calcium in the blood was much lower in both *FcγRIIB^−/−^* mice and their control littermates in response to LPS administration. Elevated serum blood urea nitrogen (BUN) and creatinine were observed in mice exposed to LPS [44].

In this study, the deletion of *FcγRIIB* resulted in mineralization defects during bone repair. LPS further delayed osteoid mineralization in both WT and *FcγRIIB^−/−^* mice. The mineralization to collagen ratio was decreased in LPS-induced *FcγRIIB^−/−^* mice. In terms of bone regeneration, we could provide the possibility that the dose of LPS used in this study directly interferes with osteoblast formation and mineralization during bone regeneration in the defects. Local LPS has potent effects on bone regeneration, including inducing greater bone loss than the otherwise comparable systemic treatment with LPS. The mineralization to collagen ratio was less in both WT and *FcγRIIB^−/−^* mice treated with LPS locally versus systemically.

This study’s disadvantage is that only a limited specificity in producing alterations in the cancellous bone was observed, which may be important in the case of studies targeting particular anatomical components of bone. Further studies on the ability of cytokine inhibitors to prevent inflammatory bone loss might shed light on the development of new therapeutic options for periodontitis and SLE patients.

In conclusion, our present study demonstrated that six-month-old *FcγRIIB^−/−^* mice exhibited osteopenia in both tibia and mandibular bones. Administration of LPS after drill-hole bone defects reduced cancellous bone volume and BMD, with downregulated osteoblast marker genes, and increased the *Tnfsf11/Tnfrsf11b* ratio. The pro-inflammatory cytokines were predominantly produced by LPS and involved in the upregulation of inflammation, resulting in impaired collagen mineralization. 

## 4. Materials and Methods

### 4.1. Animals

Male *FcγRIIB^−/−^* mice on C57BL/6 background were obtained from Dr. Silvia Bolland (NIAID, NIH, Bethesda, MD, USA). These mice were crossed with C57BL/6 females (the National Laboratory Animal Center, Mahidol University, Bangkok, Thailand) in order to generate heterozygous (*FcγRIIB*^+/*−*^) mice. Then, male and female *FcγRIIB^+/−^* mice were bred to generate *FcγRIIB^−/−^* mice and their littermate controls. They were housed at the Faculty of Medicine, Chulalongkorn University, in agreement with the use of laboratory animals that were approved by the Institutional Animal Care and Use Committee (IACUC) at the Faculty of Medicine, Chulalongkorn University. The experiments were conducted according to the guidelines of the Animal Research: Reporting In Vivo Experiments (ARRIVE). 

All experiments were performed in accordance with the Guide for the Care and Use of Laboratory Animals (eight edition), National Research Council. This study followed the ARRIVE guidelines for animal studies. The mice were maintained in a pyrogen-free environment at 24 ± 2 °C and had a standard 12-h light/12-h dark cycle. They had ad libitum access to food (C.P. Mice Feed, Perfect Companion Group Co., Ltd., C57BL/6 females (the National Laboratory Animal Center, Mahidol University, Bangkok, Thailand) and water throughout the experiment. Six-month-old *FcγRIIB^−^*^/*−*^ males that had increased anti-dsDNA and B220^low^CD138^+^, markers of plasma cells that are key factors of active SLE [7], and their littermate controls were used in this study. At the end of this study, mice were anesthetized with isoflurane and sacrificed via cervical dislocation. The right tibiae and mandibles were removed and fixed in 10% neutral-buffered formalin (NBF) for microcomputed tomography (μCT) analysis and mineralization. Both femurs were frozen in liquid nitrogen and kept at −80 °C for RNA isolation and qPCR analysis.

### 4.2. DNA Isolation and Genotyping

Tail biopsies of *FcγRIIB^−/−^* and their littermate controls were collected for genotyping via PCR. Briefly, the mouse tail was cut off approximately at a length of 3 mm and put in a microcentrifuge tube. Genomic DNA of the tail snips were isolated by adding 500 µL of tail lysis buffer master mix (100 mM Tris-HCl, pH 8.5, 5 mM EDTA, 0.2% sodium dodecyl sulfate, 200 mM NaCl) followed by 100 µg/mL of proteinase K. Then, the tail samples were incubated at 55 °C for 2 h. After that, the samples were washed with 500 µL isopropanol and centrifuged at 10,000 rpm for 10 min. In each tube, the pellet was kept but the supernatant was decanted before adding 300 µL of distilled water and incubating at 55 °C for 15 min. 

The *FcγRIIB^−/−^* and WT genotypes were identified by PCR using 3 specific primers (P): P1 (5′-AAGGCTGTGGTCAAACTCGAGCC-3′), P2 (5′-CTCGTGCTTTACGGTATCGCC-3′), and P3 (5′-TTGACTGTGGCCTTAAACGTGTAG-3′).

### 4.3. Monocortical Osseous Hole Drilling and LPS Administration 

After isoflurane anesthesia, a monocortical osseous hole was created on the angular process region of the right mandibles, and a cortical hole was created on the proximal tibiae by a 1.6 and 1.0 mm diameter round burr attached to a dental drill (Krafit, Daegu, Republic of Korea), respectively. The dental drill was connected to a micromotor. 

For the local application of *E. coli* LPS (Sigma, St. Louis, MO, USA), a single dose of 500 µg on a 2 × 2 mm collagen hemostat (Avitene Ultrafoam, Davol Inc, Warwick, RI, USA) was implanted in the drilled hole of mandibles and tibiae for 10 days. In the systemic administration, the mice were subcutaneously injected with 25 mg/kg of LPS on days 0, 3, 6, and 9 after surgery. The mice were sacrificed on day 10 after the drilling procedure. For the vehicle-treated group, animals were injected subcutaneously with PBS on the same day.

### 4.4. μCT Analysis

To investigate the three-dimensional bone microarchitecture in vivo, a desktop µCT35 scanner (Scanco Medical AG, Basserdorf, Switzerland) was used in accordance with the recommended guidelines [45]. Bones at the holed region of mandibles and tibiae were scanned using 7 µm voxel size, 70 kVp, 113 µA, and 800 ms integration time. One hundred and fifty transverse slices of tibiae were subjected to Gaussian filtration and segmentation using a fixed threshold at 220 of the maximal gray scale values. Two hundred slices of mandibles were scanned using a threshold at 270 of the maximal gray scale values. Several parameters of the cancellous bones were analyzed including bone volume (BV/TV, %), trabecular thickness (Tb.Th, mm), trabecular separation (Tb.Sp, mm), trabecular number (Tb.N, /mm), connectivity density (Conn.D, -), structural model index (SMI, -), and bone mineral density (BMD, mgHA/cm^3^).

### 4.5. Osteoblast Culture

Primary osteoblasts were isolated according to the methods described by Chevalier et al. [46]. After flushing the bone marrow out from long bones, the bones were minced into small pieces and placed in α-MEM medium containing 1 mg/mL collagenase type II (Worthington Biochemical Corporation, Lakewood, NJ, USA) for 2 h at 37 °C with shaking. Cells were centrifuged at 5000 rpm for 10 min. Bone fragments were transferred to 75 cm^2^ flasks containing α-MEM, 20% FBS, 100 units/mL penicillin, and 100 μg/mL streptomycin until they reached confluence. Cells were cultured in α-MEM medium containing 20% FBS, 5 mM β-glycerophosphate, 10 μM dexamethasone, and 50 μg/mL ascorbic acid for 7 days. Cells were isolated for qPCR analysis. 

### 4.6. Osteoclast Culture

Bone marrow was flushed out from long bones with α-MEM medium. Bone marrow cells were passed through a 40 μm filter. Cells were cultured in α-MEM medium containing 10% FBS, 100 units/mL penicillin, and 100 ug/mL streptomycin for 24 h. Non-adherent cells were cultured in the same medium containing 20 ng/mL M-CSF (R&D Systems, Inc., Minneapolis, MN, USA) for 2 days to generate bone marrow macrophages (BMMs). After that, BMMs were cultured in α-MEM medium containing 20 ng/mL M-CSF and 3.3 ng/mL RANKL (R&D Systems, Inc., Minneapolis, MN, USA) for 6 days. Cells were isolated for qPCR analysis.

### 4.7. qPCR Analysis

Total RNA from distal metaphysis of femurs was isolated using Trizol reagent (Invitrogen, Carlsbad, CA, USA). The extracted RNA samples were cleaned up and purified using a RNeasy Mini kit (Qiagen, Hilden, Germany), and the quantity of RNA samples was measured by NanoDrop 1000 (Thermo Fisher Scientific, Waltham, MA, USA). The total RNA was converted into cDNA using SuperScript VILO (Invitrogen, Carlsbad, CA, USA). The qPCR master mix was performed using forward and reverse primers and Luna Universal qPCR master mix (New England Biolabs, Ipswich, MA, USA) and run at 60 °C for 40 cycles using CFX96^TM^ Optics Module (Bio-Rad, Hercules, CA, USA). For data quantification, *Gapdh* was used as an internal control. A list of primer sequences is provided in Supplementary Table S1.

### 4.8. Mineralization Assay

Proximal metaphases of the right tibiae were decalcified in 10% ethylenediaminetetraacetic acid (EDTA) with pH 7.4 and embedded in paraffin. The decalcified samples were cut at 5 μm thickness on a microtome (Leica 2065, Wetzlar, Germany). A section from each bone specimen was stained with aniline blue for the quantitative evaluation of collagen accumulation, as previously described [47]. The histological images were taken through Olympus BX53 with 4x objective magnification before importing and adjusting them into 106-pixel digital images using Adobe Photoshop software 23.11. In order to calculate the mineralization to collagen ratio, bone volume/tissue volume (BV/TV) from μCT analysis was divided by the percent of aniline blue-positive area. The aniline blue-positive area was determined automatically using the Magic Wand tool, Photoshop 23.11. 

### 4.9. Biochemical Assays

Serum levels of pro-inflammatory cytokines, including IL-1α, IL-1β, IL-6, IL-10, IL-12p70, IL17A, IL-23, IL-27, MCP-1, IFNβ, IFN-γ, TNF-α, and GM-CSF, were performed using a multiplex beads-based assay LEGENDplex^TM^ kit (BioLegend, San Diego, CA, USA) following the instructions provided by the manufacturer. Serum calcium, phosphorus, urea nitrogen, and creatinine levels were analyzed by colorimetric assay (Quidel, San Diego, CA, USA) as per the manufacturer’s protocols.

### 4.10. Statistical Analysis

IBM SPSS Statistics for Window Version 22 (IBM Corporation, Armonk, NY, USA) was used for statistical analyses. Data from all of the experiments were represented as mean ± SEM. Statistical differences were analyzed using two-way ANOVA followed by Fisher’s protected least significant difference test. A *p*-value of less than 0.05 was considered as a significant difference.

## Data Availability

All data generated for this study are available from the corresponding authors upon request.

## Supplementary Materials

The supporting information can be downloaded at: https://www.mdpi.com/article/10.3390/ijms242316944/s1.

## References

[1] Bultink I.E.M. (2018). Bone Disease in Connective Tissue Disease/Systemic Lupus Erythematosus. Calcif. Tissue Int..

[2] Fenton K., Fismen S., Hedberg A., Seredkina N., Fenton C., Mortensen E.S., Rekvig O.P. (2009). Anti-dsDNA antibodies promote initiation, and acquired loss of renal Dnase1 promotes progression of lupus nephritis in autoimmune (NZBxNZW)F1 mice. PLoS ONE.

[3] Niederer H.A., Clatworthy M.R., Willcocks L.C., Smith K.G. (2010). FcgammaRIIB, FcgammaRIIIB, and systemic lupus erythematosus. Ann. N. Y. Acad. Sci..

[4] Tarzi R.M., Davies K.A., Claassens J.W., Verbeek J.S., Walport M.J., Cook H.T. (2003). Both Fcgamma receptor I and Fcgamma receptor III mediate disease in accelerated nephrotoxic nephritis. Am. J. Pathol..

[5] Yilmaz-Elis A.S., Ramirez J.M., Asmawidjaja P., van der Kaa J., Mus A.M., Brem M.D., Claassens J.W., Breukel C., Brouwers C., Mangsbo S.M. (2014). FcgammaRIIb on myeloid cells rather than on B cells protects from collagen-induced arthritis. J. Immunol..

[6] Shushakova N., Skokowa J., Schulman J., Baumann U., Zwirner J., Schmidt R.E., Gessner J.E. (2002). C5a anaphylatoxin is a major regulator of activating versus inhibitory FcgammaRs in immune complex-induced lung disease. J. Clin. Investig..

[7] Saiworn W., Thim-Uam A., Visitchanakun P., Atjanasuppat K., Chantaraaumporn J., Mokdara J., Chungchatupornchai S., Pisitkun P., Leelahavanichkul A., Poolthong S. (2018). Cortical Bone Loss in a Spontaneous Murine Model of Systemic Lupus Erythematosus. Calcif. Tissue Int..

[8] Visitchanakun P., Saiworn W., Jongwattanapisan P., Leelahavanichkul A., Pisitkun P., Lotinun S. (2019). Lupus-like Disease in FcgammaRIIB(-/-) Mice Induces Osteopenia. Sci. Rep..

[9] Sakunrangsit N., Pholtaisong J., Sucharitakul J., Wanna-Udom S., Prombutara P., Pisitkun P., Leelahavanichkul A., Aporntewan C., Greenblatt M.B., Lotinun S. (2021). Identification of candidate regulators of mandibular bone loss in FcgammaRIIB(-/-) Mice. Sci. Rep..

[10] Kyogoku C., Dijstelbloem H.M., Tsuchiya N., Hatta Y., Kato H., Yamaguchi A., Fukazawa T., Jansen M.D., Hashimoto H., van de Winkel J.G. (2002). Fcgamma receptor gene polymorphisms in Japanese patients with systemic lupus erythematosus: Contribution of FCGR2B to genetic susceptibility. Arthritis Rheum..

[11] Yasuda K., Sugita N., Kobayashi T., Yamamoto K., Yoshie H. (2003). FcgammaRIIB gene polymorphisms in Japanese periodontitis patients. Genes Immun..

[12] Clatworthy M.R., Harford S.K., Mathews R.J., Smith K.G. (2014). FcgammaRIIb inhibits immune complex-induced VEGF-A production and intranodal lymphangiogenesis. Proc. Natl. Acad. Sci. USA.

[13] Uehara O., Abiko Y., Saitoh M., Miyakawa H., Nakazawa F. (2014). Lipopolysaccharide extracted from Porphyromonas gingivalis induces DNA hypermethylation of runt-related transcription factor 2 in human periodontal fibroblasts. J. Microbiol. Immunol. Infect..

[14] Soetan K.O., Olaiya C.O., Oyewole O.E. (2010). The importance of mineral elements for humans, domestic animals and plants: A review. Afr. J. Food Sci..

[15] Giannelou M., Skarlis C., Stamouli A., Antypa E., Moutsopoulos H.M., Mavragani C.P. (2020). Atherosclerosis in SLE: A potential role for serum parathormone levels. Lupus Sci. Med..

[16] Bolland S., Yim Y.S., Tus K., Wakeland E.K., Ravetch J.V. (2002). Genetic modifiers of systemic lupus erythematosus in FcgammaRIIB(-/-) mice. J. Exp. Med..

[17] Horton H.M., Chu S.Y., Ortiz E.C., Pong E., Cemerski S., Leung I.W., Jacob N., Zalevsky J., Desjarlais J.R., Stohl W. (2011). Antibody-mediated coengagement of FcgammaRIIb and B cell receptor complex suppresses humoral immunity in systemic lupus erythematosus. J. Immunol..

[18] Kobayashi T., Ito S., Yasuda K., Kuroda T., Yamamoto K., Sugita N., Tai H., Narita I., Gejyo F., Yoshie H. (2007). The combined genotypes of stimulatory and inhibitory Fc gamma receptors associated with systemic lupus erythematosus and periodontitis in Japanese adults. J. Periodontol..

[19] Newling M., Fiechter R.H., Sritharan L., Hoepel W., van Burgsteden J.A., Hak A.E., van Vollenhoven R.F., van de Sande M.G.H., Baeten D.L.P., den Dunnen J. (2020). Dysregulated Fcgamma receptor IIa-induced cytokine production in dendritic cells of lupus nephritis patients. Clin. Exp. Immunol..

[20] Andrews B.S., Eisenberg R.A., Theofilopoulos A.N., Izui S., Wilson C.B., McConahey P.J., Murphy E.D., Roths J.B., Dixon F.J. (1978). Spontaneous murine lupus-like syndromes. Clinical and immunopathological manifestations in several strains. J. Exp. Med..

[21] Kikuchi S., Santiago-Raber M.L., Amano H., Amano E., Fossati-Jimack L., Moll T., Kotzin B.L., Izui S. (2006). Contribution of NZB autoimmunity 2 to Y-linked autoimmune acceleration-induced monocytosis in association with murine systemic lupus. J. Immunol..

[22] Tucureanu M.M., Rebleanu D., Constantinescu C.A., Deleanu M., Voicu G., Butoi E., Calin M., Manduteanu I. (2018). Lipopolysaccharide-induced inflammation in monocytes/macrophages is blocked by liposomal delivery of G(i)-protein inhibitor. Int. J. Nanomed..

[23] Iyer S.S., Cheng G. (2012). Role of interleukin 10 transcriptional regulation in inflammation and autoimmune disease. Crit. Rev. Immunol..

[24] Quinn J.M., Sims N.A., Saleh H., Mirosa D., Thompson K., Bouralexis S., Walker E.C., Martin T.J., Gillespie M.T. (2008). IL-23 inhibits osteoclastogenesis indirectly through lymphocytes and is required for the maintenance of bone mass in mice. J. Immunol..

[25] Wang X.F., Wang Y.J., Li T.Y., Guo J.X., Lv F., Li C.L., Ge X.T. (2019). Colony-stimulating factor 1 receptor inhibition prevents against lipopolysaccharide -induced osteoporosis by inhibiting osteoclast formation. Biomed. Pharmacother..

[26] Cho H.H., Shin K.K., Kim Y.J., Song J.S., Kim J.M., Bae Y.C., Kim C.D., Jung J.S. (2010). NF-kappaB activation stimulates osteogenic differentiation of mesenchymal stem cells derived from human adipose tissue by increasing TAZ expression. J. Cell Physiol..

[27] Croes M., Oner F.C., Kruyt M.C., Blokhuis T.J., Bastian O., Dhert W.J., Alblas J. (2015). Proinflammatory Mediators Enhance the Osteogenesis of Human Mesenchymal Stem Cells after Lineage Commitment. PLoS ONE.

[28] Abu-Amer Y., Ross F.P., Edwards J., Teitelbaum S.L. (1997). Lipopolysaccharide-stimulated osteoclastogenesis is mediated by tumor necrosis factor via its P55 receptor. J. Clin. Investig..

[29] Liu R., Desta T., Raptis M., Darveau R.P., Graves D.T. (2008). *P. gingivalis* and *E. coli* lipopolysaccharides exhibit different systemic but similar local induction of inflammatory markers. J. Periodontol..

[30] Pussinen P.J., Vilkuna-Rautiainen T., Alfthan G., Palosuo T., Jauhiainen M., Sundvall J., Vesanen M., Mattila K., Asikainen S. (2004). Severe periodontitis enhances macrophage activation via increased serum lipopolysaccharide. Arterioscler. Thromb. Vasc. Biol..

[31] Perez-Ferro M., Serrano Del Castillo C., Sanchez-Pernaute O. (2016). Cell Membrane-bound TLR2 and TLR4: Potential Predictors of Active Systemic Lupus Erythematosus and Lupus Nephritis. J. Rheumatol..

[32] Kontoyiannis D., Kollias G. (2000). Accelerated autoimmunity and lupus nephritis in NZB mice with an engineered heterozygous deficiency in tumor necrosis factor. Eur. J. Immunol..

[33] Lee T.P., Tang S.J., Wu M.F., Song Y.C., Yu C.L., Sun K.H. (2010). Transgenic overexpression of anti-double-stranded DNA autoantibody and activation of Toll-like receptor 4 in mice induce severe systemic lupus erythematosus syndromes. J. Autoimmun..

[34] AlQranei M.S., Senbanjo L.T., Aljohani H., Hamza T., Chellaiah M.A. (2021). Lipopolysaccharide- TLR-4 Axis regulates Osteoclastogenesis independent of RANKL/RANK signaling. BMC Immunol..

[35] Koide M., Kobayashi Y., Ninomiya T., Nakamura M., Yasuda H., Arai Y., Okahashi N., Yoshinari N., Takahashi N., Udagawa N. (2013). Osteoprotegerin-deficient male mice as a model for severe alveolar bone loss: Comparison with RANKL-overexpressing transgenic male mice. Endocrinology.

[36] Ozaki Y., Koide M., Furuya Y., Ninomiya T., Yasuda H., Nakamura M., Kobayashi Y., Takahashi N., Yoshinari N., Udagawa N. (2017). Treatment of OPG-deficient mice with WP9QY, a RANKL-binding peptide, recovers alveolar bone loss by suppressing osteoclastogenesis and enhancing osteoblastogenesis. PLoS ONE.

[37] Takami M., Kim N., Rho J., Choi Y. (2002). Stimulation by toll-like receptors inhibits osteoclast differentiation. J. Immunol..

[38] Guo C., Yuan L., Wang J.G., Wang F., Yang X.K., Zhang F.H., Song J.L., Ma X.Y., Cheng Q., Song G.H. (2014). Lipopolysaccharide (LPS) induces the apoptosis and inhibits osteoblast differentiation through JNK pathway in MC3T3-E1 cells. Inflammation.

[39] Xu M.X., Sun X.X., Li W., Xie G., Yang Q., Qu Z.W., Meng Q.G. (2018). LPS at low concentration promotes the fracture healing through regulating the autophagy of osteoblasts via NF-kappaB signal pathway. Eur. Rev. Med. Pharmacol. Sci..

[40] Amamoto S., Yoshiga D., Tabe S., Kokabu S., Fujii W., Hikiji H., Tominaga K., Yoshioka I. (2022). Zoledronate and lipopolysaccharide suppress osteoblast differentiation through downregulating phosphorylation of Smad in pre-osteoblastic MC3T3-E1 cells. J. Oral Maxillofac. Surg. Med. Pathol..

[41] Zhang R., Edwards J.R., Ko S.Y., Dong S., Liu H., Oyajobi B.O., Papasian C., Deng H.W., Zhao M. (2011). Transcriptional regulation of BMP2 expression by the PTH-CREB signaling pathway in osteoblasts. PLoS ONE.

[42] Onodera S., Saito A., Hojo H., Nakamura T., Zujur D., Watanabe K., Morita N., Hasegawa D., Masaki H., Nakauchi H. (2020). Hedgehog Activation Regulates Human Osteoblastogenesis. Stem Cell Rep..

[43] Du X., Zhao D., Wang Y., Sun Z., Yu Q., Jiang H., Wang L. (2022). Low Serum Calcium Concentration in Patients With Systemic Lupus Erythematosus Accompanied by the Enhanced Peripheral Cellular Immunity. Front. Immunol..

[44] Takahashi K., Mizukami H., Kamata K., Inaba W., Kato N., Hibi C., Yagihashi S. (2012). Amelioration of acute kidney injury in lipopolysaccharide-induced systemic inflammatory response syndrome by an aldose reductase inhibitor, fidarestat. PLoS ONE.

[45] Bouxsein M.L., Boyd S.K., Christiansen B.A., Guldberg R.E., Jepsen K.J., Muller R. (2010). Guidelines for assessment of bone microstructure in rodents using micro-computed tomography. J. Bone Miner. Res..

[46] Chevalier C., Colakoglu M., Brun J., Thouverey C., Bonnet N., Ferrari S., Trajkovski M. (2021). Primary mouse osteoblast and osteoclast culturing and analysis. STAR Protoc..

[47] Hu K., Olsen B.R. (2016). Osteoblast-derived VEGF regulates osteoblast differentiation and bone formation during bone repair. J. Clin. Investig..

