# Supplementary material for: Lipopolysaccharide Impedes Bone Repair in FcγRIIB-Deficient Mice
Source: Int J Mol Sci. 2023 Nov 29;24(23):16944. doi: 10.3390/ijms242316944 (PMC10707393; doi:10.3390/ijms242316944)
Supplement: Supplementary file 1 [file ijms-24-16944-s001.zip › ijms-2634509-supplementary.pdf]

Table S1. Oligonucleotide primers for qPCR analysis.

Gene	Forward primer	Reverse primer
Sp7	CCCTTCTCAAGCACCAATGG	AAGGGTGGGTAGTCATTTGCATA
Alpl	CTTGACTGTGGTTACTGCTGATCA	GTATCCACCGAATGTGAAAACGT
Opn	CTCCAATCGTCCCTACAGTCG	CCAAGCTATCACCTCGGC
Ibsp	CCACACTTTCCACACTCTCG	CGTCGCTTTCCTTCACTTTTG
Bglap	GCTGCCCTAAAGCCAAACTCT	AGAGGACAGGGAGGATCAAGTTC
Col1a1	CCCAAGGAAAAGAAGCACGTC	ACATTAGGCGCAGGAAGGTCA
Hhip	TCGAAACGGCTACTACACCC	CTCGAACTGTCCCAGAAACT
Boc	GACGGCGGTATCCCTACTTG	GTTCCTCCCAGCTTCTGGAC
Creb5	CACCCTCAGTCAGCTTACAA	AAGGGTTGGGCTGGTAAGAA
Tnf	TTGTCTACTCCCAGGTTCTCT	GAGGTTGACTTTCTCCTGGTATG
Csf1	ACCTGTTTCCCAAGAAGAGAGCCT	AGCTGTCAACACAAGCAGCCAAAG
Csf1r	TGGCATCTGGCTTAAGGTGAA	GAATCCGCACCAGCTTGCTA
Tgfb1	GGTGGTATACTGAGACACCTTG	CCCAAGGAAAGGTAGGTGATAG
Ctsk	AGGCATTGACTCTGAAGATGCT	TCCCCACAGGAATCTCTCTG
Tnfsf11	CAAGCTCCGAGCTGGTGAAG	CCTGAACTTTGAAAGCCCCA
Tnfrsf11b	AAGAGCAAACCTTCCAGCTGC	CACGCTGCTTTCACAGAGGTC
Nfatc	TGGGAGATGGAAGCAAAGAC	ATAGAAACTGACTTGGACGGG
Gapdh	TGCACCACCAACTGCTTAG	GGATGCAGGGATGATGTTC
